# A Monoclonal Antibody to M-Phase Phosphoprotein 1/Kinesin-Like Protein KIF20B

**DOI:** 10.1089/mab.2019.0016

**Published:** 2019-08-12

**Authors:** Marvin J. Fritzler, Rachael D. Brown, Meifeng Zhang

**Affiliations:** ^1^ Department of Medicine, Cumming School of Medicine, University of Calgary, Calgary, Alberta, Canada.; ^2^ Department of Biochemistry and Microbiology, University of Victoria, Victoria, British Columbia, Canada.

**Keywords:** M-phase phosphoprotein 1 (MPP1), kinesin-like protein KIF20B, epitopes, monoclonal antibody, cell cycle

## Abstract

Kinesin-like protein KIF20B, originally named M-phase phosphoprotein 1 (MPP1), is a plus-end-directed kinesin-related protein that exhibits *in vitro* microtubule-binding and -bundling properties as well as microtubule-stimulated ATPase activity. It has been characterized as a slow molecular motor that moves toward the plus-end of microtubules. Human autoantibodies directed against KIF20B have been described in up to 25% of patients with idiopathic ataxia and less commonly in other neuropathies and autoinflammatory conditions. One of the limitations of research into the structure and function of KIF20B has been a reliable monoclonal antibody that can be used in a variety of applications. To establish a reference standard for anti-KIF20B immunoassays and facilitate studies on the role of KIF20B in developmental cell biology, we developed an IgG1 monoclonal antibody, 10C7, which reacts with the cognate KIF20B protein in Western immunoblots and in addressable laser bead immunoassays. In HEp2 cells, leptomeningeal pericytes, and transfected HEK293T cells, indirect immunofluorescence studies showed that reactivity was mainly localized to a proportion of interphase nuclei, but during metaphase, it was redistributed throughout the cytoplasm and perichromatin mass. Later in telophase/anaphase, KIF20B was localized to the stem body and midzone of the midbody. 10C7 also showed remarkable staining of a subset of cells in the cerebellum, ovary, and testis tissues. KIF20B was shown to have extensive coiled-coil domains. The monoclonal antibody, 10C7, will be of value to diagnostic laboratory scientists interested in having a reliable reference standard for anti-KIF20B immunoassays as well as cell, molecular, and developmental biology researchers.

## Background and Introduction

In 1983, Dr. P. Rao and his colleagues at the MD Anderson Hospital and Tumor Institute, University of Texas, Houston, set out to identify and characterize proteins involved in cell division as an approach to elucidate the pathophysiology of malignancies.^(1)^ Their approach included raising murine monoclonal antibodies that reacted with proteins extracted from synchronized mitotic cells.^(1)^ Confirmation of monoclonal antibody targets in dividing cells relied primarily on indirect immunofluorescence (IIF) reactivity to human mitotic cells. One monoclonal antibody named MPM2 was of interest due to its reactivity with more than forty 55–220-kDa M-phase proteins, including three with molecular masses of 182, 118, and 70 kDa.^(2)^ All three of these were thought to be phosphoproteins, as evidenced by P^32^ metabolic labeling and lability after alkaline phosphatase treatment.^(2)^

In 1994, Westendorf et al. reported that two partial-length cDNAs encoding two proteins, which they named M-phase phosphoprotein 1 (MPP1) and M-phase phosphoprotein 2 (MPP2), were targets of the MPM2 monoclonal antibody.^(2)^ The deduced MPPl and MPP2 amino acid sequences were unrelated to any previously described proteins, and the ubiquitous phospho-epitope bound by the MPM2 monoclonal antibody was identified as a phosphothreonine consensus sequence Leu-Thr-Pro-Leu-Lys (LTPLK).^(2)^ This seminal article was followed by descriptions of other MPM2 targets, which were expressed during interphase, phosphorylated during mitotic induction, shared a phosphorylated epitope (present in mitosis-specific antigens in a wide range of species), and contained the aforementioned phosphothreonine consensus sequence.^(1–5)^ The other MPM2-reactive proteins included cdc25,^(6)^ microtubule-associated protein-4,^(7)^ topoisomerase IIa,^(8)^ an M-phase-specific Hl kinase,^(9)^ and MPP10.^(4,10)^

In 1996, MPP1 was reported to be specifically phosphorylated at the G2/M transition of the cell cycle,^(4)^ and in a subsequent study by Abaza et al.,^(11)^ it was identified as a plus-end-directed kinesin-related protein that exhibited *in vitro* microtubule-binding and microtubule-bundling properties as well as microtubule-stimulated ATPase activity. The importance of MPP1 in cytokinesis was strengthened when it was demonstrated that suppression of MPP1 by RNA interference induced failure of cell division late in cytokinesis.^(11)^

In 1999, when our laboratory was interested in identifying autoantibody biomarkers for a broad range of neurological diseases, our attention was drawn to a 66-year-old male who developed progressive ataxia without evidence of malignancy. His serum produced a distinctive IIF staining pattern on HEp-2 cells and unique bands on Western blots of cell extracts.^(12)^ When this serum was used to immunoscreen a HeLa cell cDNA expression library, a single reactive clone was isolated with a DNA sequence having high (>90%) similarity with MPP1.^(12)^ Furthermore, studies of serum samples from other patients with idiopathic ataxia, including some obtained from the National Neurological Specimen Bank at the University of California (Los Angeles), showed that up to 25% had antibodies that immunoprecipitated the *in vitro* transcribed and translated (TnT) recombinant MPP1.^(12)^ Four other patients bearing anti-MPP1 antibodies in this study had sensory neuropathy, two had chronic inflammatory demyelinating polyneuropathy, and one had multifocal motor neuropathy. A common, but not universal, feature of these serum samples was reactivity with interphase nuclei of HEp-2 and HeLa cells and staining of the cytoplasm of cerebellar cells (Fritzler MJ, unpublished observations). Using scleroderma serum and a similar approach of immunoscreening a HeLa cell expression library, in 2001, Kamimoto et al. detected a protein they identified as a kinesin-related protein M1 (KRMP1), which is identical to the carboxy-terminal domain of MPP1 (NCBI Accession: NP_001271188.1; Entrez Gene 23229).^(13)^

Eventually, the nomenclature of MPP1 was changed to Kinesin-like protein KIF20B and is the nomenclature adhered to throughout the rest of this article. Outside of studies of KIF20B molecular partners and ligands, an in-depth study of KIF20B/MPP1 as an autoantibody target in human diseases has not been published, although there is a single report describing KIF20B autoantibodies in paroxysmal nocturnal hemoglobinuria.^(14)^ To facilitate further research of biomarkers in autoimmune diseases, we now report the features of a monoclonal antibody directed against KIF20B. This article extends our previous clinical studies^(12,15)^ and provides information on a monoclonal antibody that can be used for future research.

## Methods

### Generation of monoclonal antibodies

Monoclonal antibody targets on KIF20B were identified based on predictive and analytic algorithms (ImmunoPrecise Antibodies Ltd., Victoria, BC, Canada) used as a strategy to synthesize the respective immunogenic peptides. These peptides were covalently coupled to keyhole limpet hemocyanin through an N-terminal cysteine for immunization protocols to produce monoclonal antibodies (ImmunoPrecise Antibodies Ltd.). Forty-eight candidate monoclonal supernatants were initially screened by ELISA for IgG and IgM reactivity and then further screened for IgG reactivity by an addressable laser bead immunoassay (ALBIA: described below) and by IIF on HEp-2 cells (Inova Diagnostics, San Diego, CA) and cryopreserved tissue sections (Inova Diagnostics; MeDiCa, Encinitas, CA). Based on these screening protocols, one monoclonal antibody (10C7) was selected for detailed analysis because it was an IgG1 isotype, demonstrated the highest reactivity in the ALBIA and Western blots of cell lysates, and demonstrated specific IIF staining of tissues and tissue culture cells (data in results).

### Transfected and nontransfected cell lysates

HEK293T cells (American Type Culture Collection, Cedarlane, Burlington, ON, Canada) were seeded in culture plates (Nunc UpCell Surface 10 cm; Thermo Fisher Scientific, Waltham, MA) and incubated for 1 day to enhance attachment before transfection with the full-length human *KIF20B* cDNA (NM_016195) tagged with green fluorescent protein (GFP) obtained from Origene (Catalog No.: RG215061; Rockville, MD). It was determined that at 48 hours after transfection, HEK293T cells most efficiently overexpressed KIF20B, as determined by IIF. Hence, subsequent cell lysates were prepared by first washing cells with cold phosphate-buffered saline (PBS) and then harvesting cells on ice using cold NETN buffer (150 mM NaCl, 1 mM EDTA, 50 mM Tris-HCl (pH 7.4), 1% Nonidet P-40/Tergitol, and protease inhibitor (Complete Mini, Roche, Indianapolis, IN), and phosphatase inhibitor (PhosSTOP, Roche, Indianapolis, IN). Lysates were centrifuged for 15 minutes at 11,000 rpm at 4°C, aliquoted, and stored at −80°C. Untransfected HeLa cells (American Type Culture Collection) were also cultured and lysates prepared as described above.

### Western blot

Lysates of transfected HEK293T and HeLa cells fractionated on 4%–12% gradient gels (Thermo Scientific, Rockford, IL) and after SDS-PAGE^(16)^ were transferred to nitrocellulose membranes using an iBlot 2 gel transfer device (Thermo Scientific). The membranes were blocked with 5% (wt/vol) nonfat dry milk in 0.1% (vol/vol) Tween 20–Tris-buffered saline (TBS-T; blocking buffer) for 2 hours at room temperature or overnight at 4°C. Human and monoclonal antibodies were diluted with blocking buffer and overlaid on membrane strips with overnight incubation at 4°C with gentle rocking. After washes in several changes of 0.1% (vol/vol) TBS-T, the membrane strips were incubated with peroxidase-conjugated goat anti-mouse IgG (H+L) or anti-human IgG (H+L) (AffiniPure: Jackson ImmunoResearch, West Grove, PA) diluted 1:100,000 in blocking buffer for 1 hour at room temperature. After washes in TBS-T, membranes were incubated in a solution of SuperSignal West Pico PLUS substrate (Thermo Scientific) according to the manufacturer's instructions and the signals detected using the LI-COR C-Digit Blot Scanner (LI-COR, Lincoln, NE).

### Human anti-KIF20B antibodies

Serum samples from three index patients with high titers of anti-KIF20B, as previously determined in a TnT recombinant immunoprecipitation assay^(12)^ and ALBIA (described below), were selected to serve as reference controls in our evaluation of monoclonal antibodies. Using standard operating procedures, serum was collected and biobanked at a central laboratory (Mitogen Advanced Diagnostics Laboratory, Calgary, AB, Canada) where aliquots were stored at −80°C until needed. These serum samples were extensively tested to rule out autoantibodies to other intracellular targets and extractable nuclear antigens by ALBIA (FIDIS Connective 13; TheraDiag, Paris, France), line immunoassay (Euroline Systemic Sclerosis; Systemic Lupus Profile, Euroimmun GmbH, Luebeck Germany), or chemiluminescence immunoassay (QUANTA Flash CIA; Inova Diagnostics).^(17)^ The analytes included in these assays were dsDNA, Sm, U1-RNP, SS-A/Ro60, Ro52/TRIM21, SSB/La, ribosomal P, histones, proliferating cell nuclear antigen, Jo-1 (histidyl-tRNA synthetase), centromere protein (CENP)-A, CENP-B, topo-I/Scl-70, RNAPIII, PM/Scl (PM75 and/or PM100), Ro52/TRIM21, platelet-derived growth factor receptor (PDGFR), Ku, Th/To, NOR90/hUBF (human upstream binding factor), and fibrillarin (U3RNP). If the anti-KIF20B human serum samples were negative on these assays, they were deemed to be monospecific for anti-KIF20B and were used as reference serum samples for epitope mapping and other assays.

### KIF20B immunoassay

The monospecific human anti-KIF20B-positive serum samples described above were used to establish an ALBIA on the MAGPIX platform (Luminex Corp., Austin, TX) adapted from previously described protocols.^(18,19)^ Antigens coupled to beads (MagPlex microspheres; Luminex Corp.) included the full-length recombinant human KIF20B containing a GFP tag in HEK293T cell extracts described above, recombinant partial-length protein (corresponding to the KIF20B amino acid sequence 1671–1780; Abnova, Taipei, Taiwan), and peptides representing synthetic peptides (QPKRAKRKLYTSEISS) used to generate KIF20B monoclonal antibodies (ImmunoPrecise Antibodies Ltd.). Antigens were coupled to beads as previously described.^(18)^ Briefly, 2 μL of suspended beads in solution (Luminex Corp.), 38 μL of sample diluent (Inova Diagnostics), and 10 μL of serum samples diluted 1/100 were pipetted into wells of light-tight microtiter plates (Luminex Corp.). The plate was covered to limit exposure of beads to ambient light and incubated on a shaker for 30 minutes at room temperature. After the beads were washed to remove unbound components, 50 μL of diluted, phycoerythrin-conjugated secondary antibody (goat anti-human or anti-mouse, as appropriate) (Jackson ImmunoResearch) was added and incubated with gentle agitation at 600 rpm for 30 minutes at room temperature in the dark. Positive and negative control samples were included in each run, and plates were analyzed by using the Luminex 100 or MagPix^®^ system (Luminex Corp.). Data were expressed as the median fluorescence intensity (MFI) and positivity was defined as titers >500 MFI for lysates, and for synthetic peptides, positivity was defined as titers >250 MFI, three standard deviations above the values observed in apparently healthy control samples.

### IIF and colocalization

IIF on HEp-2 substrate (HEp-2; Inova Diagnostics), human leptomeningeal pericytes, and monkey tissue substrates (Medica, Carlsbad, CA) were prepared using conventional protocols and read on a Zeiss Universal microscope fitted with a 150-W ultraviolet light source or on an automated imaging system (NOVA View; Inova Diagnostics). Monoclonal 10C7 and patient serum as primary antibodies were diluted in PBS and Alexa Fluor 488 goat anti-mouse IgG (H + L chains) (Thermo Fisher Scientific, Waltham, MA) was used as the secondary antibody. In parallel studies of the index, human serum sample or rabbit antibody Alexa Fluor 488 goat anti-human IgG (H+L; Thermo Fisher Scientific) or Alexa Fluor 568 goat anti-rabbit IgG (H+L; Thermo Fisher Scientific) diluted 1:500 in PBS was used as the secondary antibody.

### Epitope mapping

Reactivity of the 10C7 monoclonal antibody directed against KIF20B was subjected to epitope mapping on sequential overlapping peptides on a solid-phase matrix (PEPperPRINT GmbH, Heidelberg, Germany). As controls, a murine monoclonal directed to golgin-97,^(20,21)^ monospecific control human anti-KIF20B serum from a patient, and serum from a healthy individual were also used for this epitope analysis. The sequences of KIF20B (Entrez Gene 23229) were elongated with neutral GSGSGSG linkers at the C- and N-termini to avoid truncated peptides. The elongated antigen sequences were translated into 15 amino acid peptides with a peptide–peptide overlap of 12 amino acids, resulting in 608 different peptides printed in duplicate (1216 peptide spots in all) using solid-phase peptide arrays (PEPperPRINT, Heidelberg, Germany).^(22,23)^ The corresponding peptide microarrays were further framed by Flag and HA control peptides (100 spots each). The candidate KIF20B serum samples were diluted in incubation buffer (PBS, pH 7.4, with 0.05% Tween 20) and 10% MB-070 blocking buffer (Rockland Immunochemicals, Inc., Limerick, PA) and incubated with peptide microarrays for 16 hours at 4°C and swirling at 500 rpm. Unbound antibodies were removed by washing the arrays with washing buffer. Prestaining of one of the peptide arrays was done with F(ab’)2 goat anti-human IgG (H+L) antibody conjugated to DyLight680 (Thermo Fisher Scientific) at a dilution of 1:5000 to investigate background interactions with antigen-derived peptides.

HA and Flag control peptides framing the peptide arrays were finally stained with monoclonal anti-HA (12CA5)-DyLight 680 and monoclonal anti-FLAG(M2)-DyLight 800 (Thermo Fisher Scientific) in an incubation buffer for 1 hour at RT and dilution of 1:1000 as internal quality control to confirm the assay quality and peptide microarray integrity (scanning intensities red/green: 7/7). Quantification of spot intensities and peptide annotation utilized the LI-COR Odyssey Imaging System (scanning offset 1 mm, resolution 21 μm, and scanning intensities in green/red of 7/7) and PepSlide^®^ Analyzer. A software algorithm interpolates fluorescence intensities of each spot into raw, foreground, and background signals (see “Raw Data” tabs) and calculates the standard deviation of foreground median intensities. Based on averaged foreground median intensities, intensity maps were generated and binders in the peptide maps were highlighted by an intensity color code, with red for high and white for low spot intensities.^(22,23)^

### Coiled-coil analysis

Analysis of KIF20B for coiled-coil domains,^(24)^ a feature of a number of target autoantigens,^(25)^ was performed online with the aid of the coils server.

## Results and Discussion

**FIG. 1.**
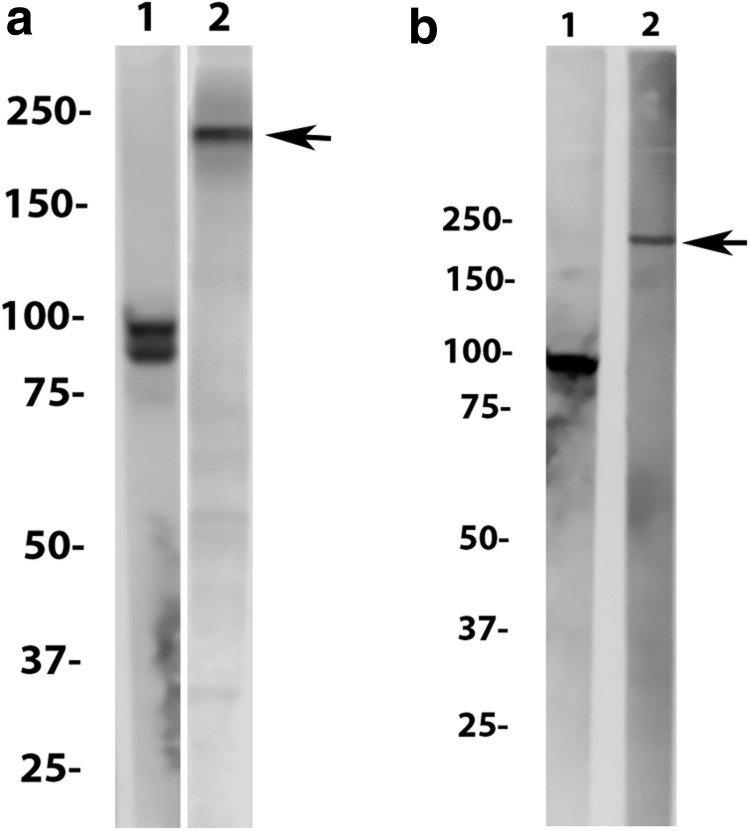
Western immunoblot of **(a)** HeLa cell lysate and **(b)** KIF20B-overexpressing HEK293T cell lysate (see Fig. 4) with a monoclonal to the ∼97-kDa Golgi complex protein golgin 97^(21)^ (lane 1) and monoclonal 10C7 anti-KIF20B (lane 2). KIF20B is identified as a ∼2325-kDa protein (arrows). Conditions: **(a)** 50 μg of Hela cell lysate loaded on gel and probed with monoclonal 10C7 diluted 1:4. **(b)** 80 μg of KIF20B-overexpressing HEK293T cell lysate loaded on gel and probed with monoclonal 10C7 diluted 1:4.

Western blot analysis showed that the monoclonal antibody, 10C7, bound specifically to a ∼235-kDa protein in HeLa cell lysates (Fig. 1a) and KIF20B-overexpressing HEK293T cell lysates (Fig. 1b). Confirmation that 10C7 indeed reacted with the immunizing peptide was established by retesting its reactivity using epitope mapping protocols that measured its binding to overlapping peptides representing the full length of KIF20B (Supplementary Fig. S1). In this study, the most predominant binding was to the cognate immunizing peptide (KLYTSEISSPIDISG: amino acids 1779–1790) near the carboxy terminus, although much weaker reactivity to a peptide (ARTQNLKADLQRKEE: amino acids 1269–1282) upstream toward the amino terminus was also observed. By comparison, the primary epitopes of the human anti-KIF20B serum samples were to 548–ETKLLDEDLDKTL–560 and 1691–VKKEQKVAIRPSSKK–1706 near the carboxyl terminus (data not shown). Next, reactivity of 10C7 with purified KIF20B was confirmed by ALBIA (Table 1) where it showed very high binding, exceeding the binding of the index human anti-KIF20b by more than fivefold.

**Table 1.** Reactivity of Monoclonal 10C7 in an Addressable Laser Bead Immunoassay Compared with Index Human Serum Samples and Control Monoclonal Anti-Golgin97

*Sample*	*Reactivity recombinant protein (MFI)*	*Reactivity immunizing peptide (MFI)*	*Reactivity* in vitro *overexpressed KIF20B*^a^
10C7 anti-KIF20B	17953	24038	ND
Murine monoclonal anti-Golgi (4B6 anti-golgin97)	122	98	ND
Human1 anti-KIF20B	3396	407	2608
Human2 anti-KIF20B	145	171	2721
Human3 anti-KIF20B	809	669	7745
Human disease control	276	542	208
Normal human serum	233	198	89

^a^ Lysates of HEK293T cells transfected with and overexpressing KIF20B.

ND, not done because the overexpressed KIF20B includes a GFP tag and is coupled to the ALBIA beads through a mouse anti-tGFP ligand, resulting in nonspecific reactivity to the PE-conjugated anti-mouse IgG secondary antibody.

ALBIA, addressable laser bead immunoassay; GFP, green fluorescent protein; MFI, median fluorescence intensity; PE, phycoerythrin.

**FIG. 2.**
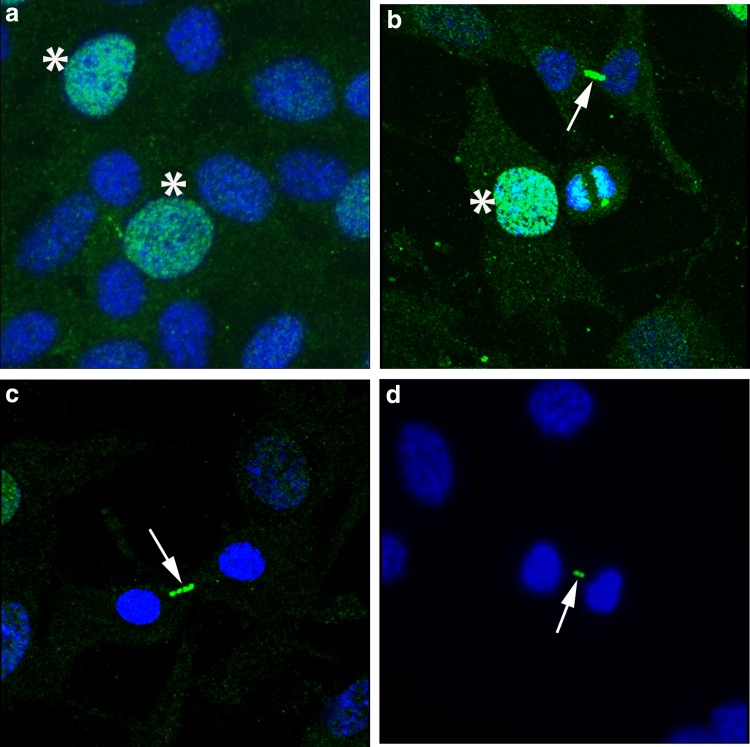
Indirect immunofluorescence of 10C7 monoclonal antibody to KIF20B on HEp-2 cells. Nuclei (**a, b** asterisks) of ∼20% of cells were stained, with intense staining of the intracellular bridge and midbody region (arrows in **b–d**). Faint speckling of cytoplasm was consistently observed and the interchromatin mass of late M-phase cells **(b)** is also stained (spinning disk confocal, original magnification 600 × ).

Having confirmed the reactivity of 10C7 to a 235-kDa target and the cognate immunizing peptide, our attention turned to reactivity of 10C7 to tissue culture cells and tissues. First, IIF staining of commercially available HEp-2 cells (Inova) showed pleomorphic staining of nuclei in ∼20% of cells and intense staining of the intracellular bridge and midbody region (Fig. 2). In addition, faint, cytoplasmic speckled staining was consistently observed, the interchromatin mass of late M-phase cells was also stained. Staining of fixed/permeabilized HeLa cells also demonstrated pleomorphic nuclear staining and intense staining of a portion of the intracellular bridge (Supplementary Fig. S2).

**FIG. 3.**
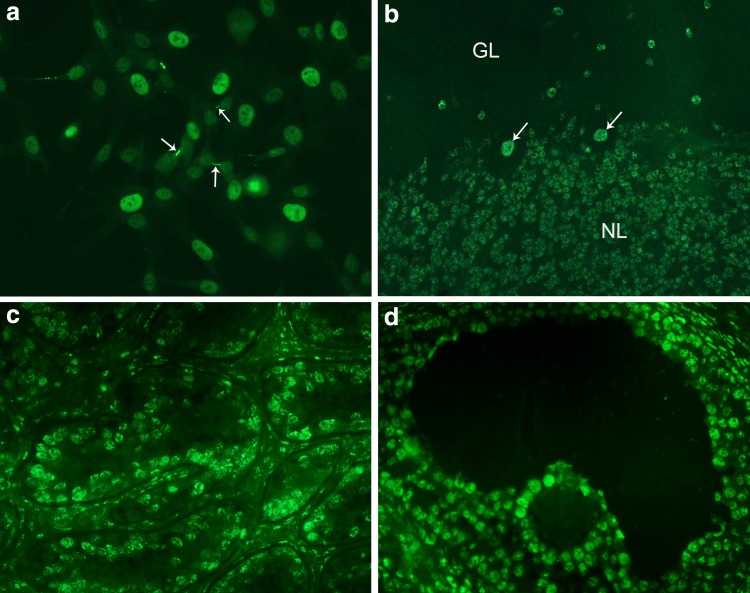
Monoclonal 10C7 directed to KIF20B (1:10 diluted in PBS) reacts with **(a)** nuclei and the intercellular bridge (arrows) of human leptomeningeal pericyte cells (cells fixed and permeabilized with 4% PFA/0.2% Triton X-100); **(b)** granular layer (GL) and Purkinje cells (arrows) of monkey cerebellum; and **(c)** nuclei in monkey testis and **(d)** ovary tissues. Goat anti-mouse IgG Alexa488 diluted at 1:500 in PBS was used as the secondary antibody. PBS, phosphate-buffered saline.

**FIG. 4.**
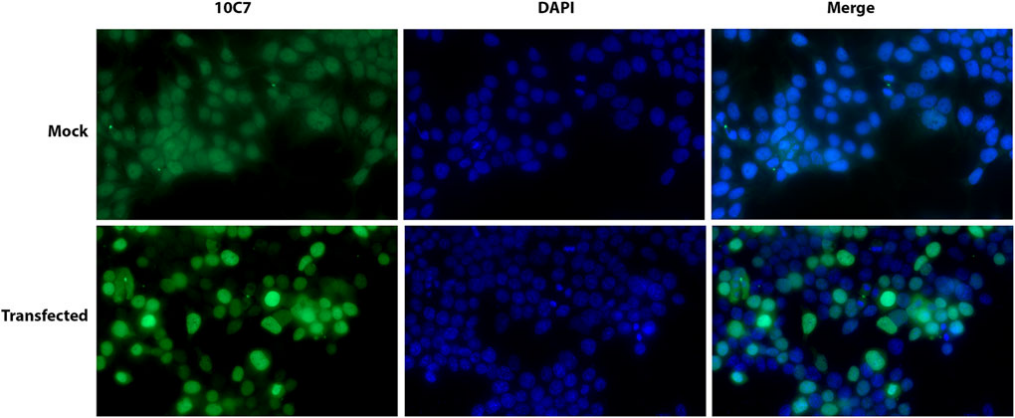
HEK293T cells were transfected with full-length human KIF20B cDNA (transfected) or exposed to the transfection carrier only (mock), and after incubation, to allow overexpression of KIF20B cDNA (OriGene Technologies, Inc.), they were fixed and permeabilized with 3% PFA/0.2% Triton x-100 and stained with monoclonal 10C7 anti-KIF20B (diluted at 1:10 in PBS) and DAPI (visualize cell nuclei). KIF20B is overexpressed in transfected cells compared with mock treated cells and the typical, variable, speckled cell staining was observed in ∼50% of cells (merge).

**FIG. 5.**
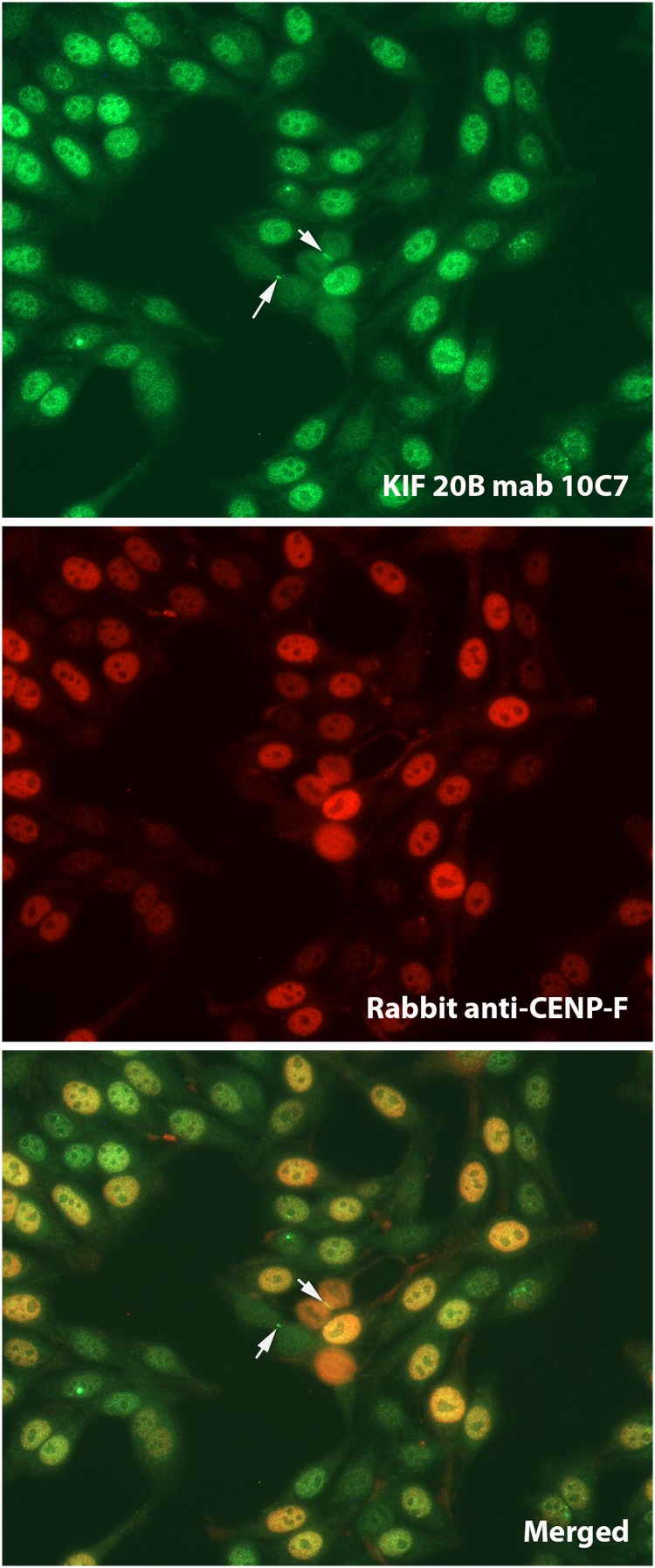
Monoclonal 10C7 anti-KIF20B colocalization (diluted at 1:10 in PBS) with rabbit anti-CENP-F (mitosin, diluted at 1:1000 in PBS) on commercially available HEp-2 cells (ImmunoConcepts). At telophase, KIF20B is concentrated within the intracellular bridge at either side of the midbody (arrows). CENP-F, centromere protein F.

IIF staining of human leptomeningeal cells (Fig. 3a) was similar to that observed in HEp-2 cells. These IIF findings on HEp-2 and leptomeningeal cells are consistent with previously published descriptions of IIF staining in dividing cells, which showed that MPP1/KIF20B was mainly localized to interphase nuclei, but during metaphase, it was redistributed throughout the cytoplasm, and later in telophase/anaphase also, it localized to the midzone of the midbody.^(11)^ In the monkey cerebellum, we also observed staining of cells in both the granular and molecular layers, as well as Purkinje cells (Fig. 3b). When other monkey tissues were tested for reactivity, the most remarkable observation was staining of nuclei in the ovary and testis (Fig. 3c, d), whereas reactivity of other tissues (i.e., liver, stomach, and kidney) showed only weak apparently nonspecific staining. We are not aware of other published studies reporting IIF reactivity of tissue and organs with monoclonal antibodies to MPP1/KIF20B. In our initial report,^(12)^ a monospecific human antibody also produced a speckled IIF pattern of interphase cells, but staining in the midbody/stem body was not observed. In addition, reactivity with nuclei of unidentified cerebellar cells, but not Purkinje cells, was demonstrated in that earlier report. These differences of staining between the monoclonal 10C7 and the human serum samples may be due to differences in epitopes bound by the respective antibodies (detailed above) where it was clearly shown that the main epitopes bound by human anti-KIF20B serum samples did not overlap with the epitope bound by the monoclonal 10C7. Importantly, not all tissue culture cells produced this IIF pattern of staining. As one example, human embryonic kidney cells (HEK293T) showed only weak nonspecific staining (Fig. 5), although after transfection with full-length KIF20B cDNA, a typical staining pattern was observed (Fig. 4).

Next, we set out to determine if the cell cycle staining of 10C7 was unique or identical to other cell cycle components such as CENP-F, a centromere-related protein that also has a cell cycle staining pattern.^(26,27)^ CENP-F (mitosin) is a nuclear matrix protein that gradually accumulates during the cell cycle until it reaches peak levels in G2- and M-phase cells and is rapidly degraded upon completion of mitosis. However, when colocalization studies were preformed (Fig. 5), there was some overlap of staining of interphase and mitotic cells, but the anti-CENP-F serum samples did not demonstrate the same intense staining of the midbody/stem body as that of 10C7. Although previously reported,^(11)^ the significance of localization of KIF20B to the stem body of dividing cells is not fully known, although there is evidence that KIF20B plays an important role in cytokinetic furrowing and mediation of cell abscission,^(28,29)^ but not midbody assembly.^(30)^ Notably, disruption of abscission in a loss-of-function KIF20B mutant did not result in binucleated or multinucleated cells, but (curiously) apoptosis.^(28)^ Furthermore, the importance of KIF20B in brain and neural development has been emphasized by its effects on regulating neuron polarization, axon guidance, and dendrite branching and outgrowth.^(30,31)^ Taken together, there is evidence that through recruitment of some proteins and dispersal of others, KIF20B plays a key role in formation of microtubule constriction sites.^(30)^

**FIG. 6.**
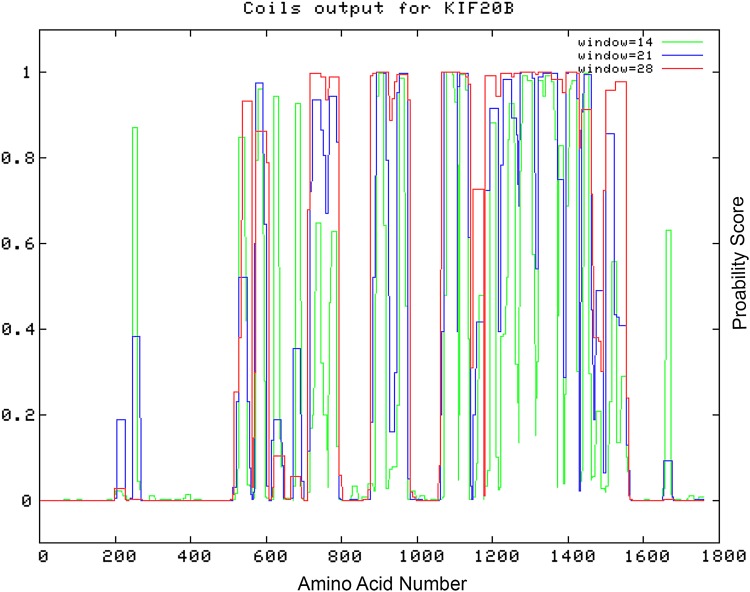
MPP1/KIF20B is characterized by extensive coiled-coil regions except for two regions (amino acids 1–200 and approximately 275–560) at the carboxyl terminus and then a similar, but smaller, region at the amino terminus (aa 1575–1800). Note that the epitopes reactive with 10C7 (amino acids 1772–1787, Uniprot: Q96Q89) and the human anti-KIF20B serum samples (amino acids 548–560; 1691–1706; carboxy terminus) are not localized to coiled-coil regions of KIF20B. MPP1, M-phase phosphoprotein 1.

Previous studies have shown that many target autoantigens in a number of cell compartments, especially those involved in organelle movement, are characterized as containing extensive coiled-coil motifs.^(25,32)^ For example, in the endosomal compartment, two coiled-coil autoantigens are early endosomal protein EEA1 (180 kDa)^(33)^ and CLIP-170 (170 kDa).^(34)^ Certain centrosomal autoantigens were also identified as coiled-coil-rich proteins, including pericentrin, a 220-kDa protein^(35)^; ninein, a protein with alternatively spliced products of 245 and 249 kDa^(36)^; Cep250 (250 kDa); and Cep110 (110 kDa).^(37)^ Centromere autoantigens have been described, but the most interesting protein related to this discussion is CENP-F,^(38)^ a high-molecular-weight protein (∼400 kDa), which has similar, but (as shown above) not identical, cell cycle IIF staining patterns as monoclonal anti-KIF20B antibodies. NuMA is another large coiled-coil protein located at the mitotic spindle pole and is the most common target autoantigen in serum samples with mitotic spindle apparatus staining.^(39)^ Nonmuscle myosin (∼200 kDa) is a cytoskeletal autoantigen^(40)^ qualified in the same group of high-molecular-weight and coiled-coil-rich autoantigens. Hence, it is intriguing that KIF20B is also characterized by extensive coiled-coil regions (Fig. 6). However, the major epitopes bound by monoclonal 10C7 or human anti-KIF20C serum samples are not localized to these coiled-coil regions. This is reminiscent of a recently identified autoantigen, bicaudal D2 (BICD2), which also has significant coiled-coil regions, but the major B cell epitope is a region of the protein devoid of coiled-coil motifs.^(41)^ In a seminal publication, the first 302 amino acids of MPP1 were reported as having a coiled-coil domain.^(2)^ Our study has extended this finding to the entire MPP1/KIF20B protein.

## Summary

Our interest in KIF20B was piqued when we discovered that it was a target autoantigen in up to 25% of patients with idiopathic ataxia and in some patients with demyelinating polyneuropathy.^(12,15)^ Given the importance of KIF20B in neural development, it is interesting to consider that these antibodies may be biomarkers for ataxia and/or other neuropathies. The study of KIF20B in cell physiology and anti-KIF20B as a target in some autoinflammatory diseases will be facilitated by the availability of a new monoclonal anti-KIF20B antibody, 10C7, described herein.

## Supplementary Material

Supplemental data

## References

[1] Davis FM, Tsao TY, Fowler SK, and Rao PN: Monoclonal antibodies to mitotic cells. Proc Natl Acad Sci U S A 1983;80:2926–2930 6574461 10.1073/pnas.80.10.2926 PMC393946

[2] Westendorf JM, Rao PN, and Gerace L: Cloning of cDNAs for M-phase phosphoproteins recognized by the MPM2 monoclonal antibody and determination of the phosphorylated epitope. Proc Natl Acad Sci U S A 1994;91:714–718 8290587 10.1073/pnas.91.2.714 PMC43019

[3] Zhao L, Kuang J, Adlakha RC, and Rao PN: Threonine phosphorylation is associated with mitosis in HeLa cells. FEBS Lett 1989;249:389–395 2500366 10.1016/0014-5793(89)80665-9

[4] Matsumoto-Taniura N, Priollet F, Monroe R, Gerace L, and Westendorf JM: Identification of novel M phase phosphoproteins by expression cloning. Mol Biol Cell 1996;7:1455–1469 8885239 10.1091/mbc.7.9.1455 PMC275994

[5] Engle DB, Doonan JH, and Morris NR: Cell-cycle modulation of MPM-2 specific spindle pole body phosphorylation in Aspergillus nidulans. Cell Motil Cytoskel 1988;10:432–437 10.1002/cm.970100310 3052873

[6] Kuang J, Ashorn CL, Gonzales-Kuyvenhoven M, and Penkala JE: cdc25 is one of the MPM-2 antigens involved in the activation of maturation-promoting factor. Mol Biol Cell 1994;5:135–145 8019000 10.1091/mbc.5.2.135 PMC301020

[7] Vandre DD, Centonze VE, Peloquin J, Tombes RM, and Borisy GG: Proteins of the mammalian mitotic spindle: Phosphorylation/dephosphorylation of MAP-4 during mitosis. J Cell Sci 1991;98:577–588 1860904 10.1242/jcs.98.4.577

[8] Taagepera S, Rao PN, Drake FH, and Gorbsky GJ: DNA topoisomerase IIα is the major chromosome protein recognized by the mitotic phosphoprotein antibody MPM-2. Proc Natl Acad Sci U S A 1993;90:8407–8411 7690961 10.1073/pnas.90.18.8407 PMC47365

[9] Kuang J, Penkala JE, Wright DA, Saunders GF, and Rao PN: A novel M phase-specific H1 kinase recognized by the mitosis-specific monoclonal antibody MPM-2. Dev Biol 1991;144:54–64 1995402 10.1016/0012-1606(91)90478-l

[10] Westendorf JM, Konstantinov KN, Wormsley S, Shu MD, Matsumoto-Taniura N, Pirollet F, Klier FG, Gerace L, and Baserga SJ: M phase phosphoprotein 10 is a human U3 small nucleolar ribonucleoprotein component. Mol Biol Cell 1998;9:437–449 9450966 10.1091/mbc.9.2.437 PMC25272

[11] Abaza A, Soleilhac JM, Westendorf J, Piel M, Crevel I, Roux A, and Pirollet F: M phase phosphoprotein 1 is a human plus-end-directed kinesin-related protein required for cytokinesis. J Biol Chem 2003;278:27844–27852 12740395 10.1074/jbc.M304522200 PMC2652640

[12] Fritzler MJ, Kerfoot SM, Feasby TE, Zochodne DW, Westendorf JM, Dalmau JO, and Chan EK: Autoantibodies from patients with idiopathic ataxia bind to M-phase phosphoprotein 1 (MPP-1). J Invest Med 2000;48:28–39 10695267

[13] Kamimoto T, Zama T, Aoki R, Muro Y, and Hagiwara M: Identification of a novel kinesin-related protein, KRMP1, as a target for mitotic peptidyl-prolyl isomerase Pin1. J Biol Chem 2001;276:37520–37528 11470801 10.1074/jbc.M106207200

[14] Alahmad A, Preuss KD, Schenk J, Füreder W, Schrezenmeier H, Müller-Lantzsch N, Schubert J, and Pfreundschuh M: Desmoplakin and *KIF20B* as target antigens in patients with paroxysmal nocturnal haemoglobinuria. Br J Haematol 2010;151:273–280 20813002 10.1111/j.1365-2141.2010.08345.x

[15] Zochodne DW, Auer R, and Fritzler MJ: Longstanding ataxic demyelinating polyneuropathy with a novel autoantibody. Neurology 2003;60:127–129 12525735 10.1212/01.wnl.0000040660.76868.3c

[16] Fritzler MJ, Lung C-C, Hamel JC, Griffith K, and Chan EKL: Molecular characterization of golgin-245: A novel Golgi complex protein containing a granin signature. J Biol Chem 1995;270:31262–31268 8537393 10.1074/jbc.270.52.31262

[17] Mahler M, Bentow C, Serra J, and Fritzler MJ: Detection of autoantibodies using chemiluminescence technologies. Immunopharmacol Immunotoxicol 2016;38:14–20 26525648 10.3109/08923973.2015.1077461 PMC4819877

[18] Selak S, Mahler M, Miyachi K, Fritzler ML, and Fritzler MJ: Identification of the B-cell epitopes of the early endosome antigen 1 (EEA1). Clin Immunol 2003;109:154–164 14597214 10.1016/s1521-6616(03)00169-4

[19] Eystathioy T, Chan EK, Takeuchi K, Mahler M, Luft LM, Zochodne DW, and Fritzler MJ: Clinical and serological associations of autoantibodies to GW bodies and a novel cytoplasmic autoantigen GW182. J Mol Med 2003;81:811–818 14598044 10.1007/s00109-003-0495-y

[20] Griffith KJ, Chan EKL, Hamel JC, Miyachi K, and Fritzler MJ: Molecular characterization of a novel 97 kDa Golgi complex autoantigen recognized by autoimmune antibodies from patients with Sjögren's syndrome. Arthritis Rheum 1997;40:1693–1702 9324025 10.1002/art.1780400920

[21] Eystathioy T, Jakymiw A, Fujita DJ, Fritzler MJ, and Chan EKL: Human autoantibodies to a novel Golgi protein golgin-67: High similarity with golgin-95/gm 130 autoantigen. J Autoimmun 1999;14:179–187 10.1006/jaut.1999.0359 10677249

[22] Beyer M, Block I, Konig K, Nesterov A, Fernandez S, Felgenhauer T, Schirwitz C, Leibe K, Bischoff RF, Breitling F, and Stadler V: A novel combinatorial approach to high-density peptide arrays. Methods Mol Biol 2009;570:309–316 19649602 10.1007/978-1-60327-394-7_16

[23] Nesterov A, Dorsam E, Cheng YC, Schirwitz C, Märkle F, Löffler F, König K, Stadler V, Bischoff R, and Breitling F: Peptide arrays with a chip. Methods Mol Biol 2010;669:109–124 20857361 10.1007/978-1-60761-845-4_9

[24] Lupas A, Van Dyke M, and Stock J: Predicting coiled coils from protein sequences. Science 1991;252:1162–1164 2031185 10.1126/science.252.5009.1162

[25] Nozawa K, Fritzler MJ, and Chan EKL: Unique and shared features of Golgi complex autoantigens. Autoimmun Rev 2005;4:35–41 15652777 10.1016/j.autrev.2004.06.002

[26] Rattner JB, Rao A, Fritzler MJ, Valencia DW, and Yen TJ: CENP-F is a .ca 400 kDa kinetochore protein that exhibits a cell-cycle dependent localization. Cell Motil Cytoskelton 1993;26:214–226 10.1002/cm.970260305 7904902

[27] Fritzler MJ, Rattner JB, Luft LM, Edworthy SM, Casiano CA, Peebles C, and Mahler M: Historical perspectives on the discovery and elucidation of autoantibodies to centromere proteins (CENP) and the emerging importance of antibodies to CENP-F. Autoimmun Rev 2010;10:194–200 20933614 10.1016/j.autrev.2010.09.025

[28] Janisch KM, Vock VM, Fleming MS, Shrestha A, Grimsley-Myers CM, Rasoul BA, Neale SA, Cupp TD, Kinchen JM, Liem KF Jr, and Dwyer ND: The vertebrate-specific Kinesin-6, Kif20b, is required for normal cytokinesis of polarized cortical stem cells and cerebral cortex size. Development 2013;140:4672–4682 24173802 10.1242/dev.093286 PMC3833427

[29] Janisch KM, and Dwyer ND Imaging and quantitative analysis of cytokinesis in developing brains of Kinesin-6 mutant mice. Methods Cell Biol 2016;131:233–252 26794517 10.1016/bs.mcb.2015.06.008

[30] Janisch KM, McNeely KC, Dardick JM, Lim SH, and Dwyer ND: Kinesin-6 KIF20B is required for efficient cytokinetic furrowing and timely abscission in human cells. Mol Biol Cell 2018;29:166–179 29167382 10.1091/mbc.E17-08-0495 PMC5909929

[31] McNeely KC, Cupp TD, Little JN, Janisch KM, Shrestha A, and Dwyer ND: Mutation of Kinesin-6 Kif20b causes defects in cortical neuron polarization and morphogenesis. Neural Dev 2017;12:5 28359322 10.1186/s13064-017-0082-5 PMC5374676

[32] Rand TA, Ginalski K, Grishin NV, and Wang X: Biochemical identification of Argonaute 2 as the sole protein required for RNA-induced silencing complex activity. Proc Natl Acad Sci U S A 2004;101:14385–14389 15452342 10.1073/pnas.0405913101 PMC521941

[33] Selak S, Schoenroth L, Senécal J-L, and Fritzler MJ: Early endosome antigen 1: An autoantigen associated with neurological diseases. J Invest Med 1999;47:311–318 10431486

[34] Griffith KJ, Ryan JP, Senécal J-L, and Fritzler MJ: The cytoplasmic linker protein CLIP-170 is a human autoantigen. Clin Exp Immunol 2002;127:533–538 11966772 10.1046/j.1365-2249.2002.01756.x PMC1906301

[35] Delaval B, and Doxsey SJ: Pericentrin in cellular function and disease. J Cell Biol 2010;188:181–190 19951897 10.1083/jcb.200908114 PMC2812529

[36] Bouckson-Castaing V, Moudjou M, Ferguson DJP, Mucklow S, Belkaid Y, Milon G, and Crocker PR: Molecular characterisation of ninein, a new coiled-coil protien of the centrome. J Cell Sci 1996;109:179–190 8834802 10.1242/jcs.109.1.179

[37] Mack GJ, Rees J, Sandblom O, Balczon R, Fritzler MJ, and Rattner JB: Autoantibodies to a group of centrosomal proteins in human autoimmune sera reactive with the centrosome. Arthritis Rheum 1998;41:551–558 9506584 10.1002/1529-0131(199803)41:3<551::AID-ART22>3.0.CO;2-X

[38] Rattner JB, Rees J, Whitehead CM, Casiano CA, Tan EM, Humbel RL, Conrad K, and Fritzler MJ: High frequency of neoplasia in patients with autoantibodies to centromere protein CENP-F. Clin Invest Med 1997;20:308–319 9336656

[39] Price CM, McCarty GA, and Pettijohn DE: NuMA protein is a human autoantigen. Arthritis Rheum 1984;27:774–779 6378210 10.1002/art.1780270708

[40] Von Mühlen CA, Chan EKL, Peebles CL, Imai H, Kiyosawa K, and Tan EM: Non-muscle myosin as target antigen for human autoantibodies in patients with hepatitis C virus-associated chronic liver diseases. Clin Exp Immunol 1995;100:67–74 7697925 10.1111/j.1365-2249.1995.tb03605.x PMC1534265

[41] Fritzler MJ, Hudson M, Choi MY, Mahler M, Wang M, Bentow C, Milo J, Baron M; and Canadian Scleroderma Research Group: Identification of Bicaudal D2: A novel autoantibody target in systemic sclerosis that shares a key epitope with CENP-A but has a distinct clinical phenotype. Autoimmun Rev 2018;17:267–275 29369808 10.1016/j.autrev.2018.01.006

