# Supplementary material for: A Monoclonal Antibody to M-Phase Phosphoprotein 1/Kinesin-Like Protein KIF20B
Source: Monoclon Antib Immunodiagn Immunother. 2019 Aug 12;38(4):162–70. doi: 10.1089/mab.2019.0016 (PMC6709729; doi:10.1089/mab.2019.0016)
Supplement: Supplemental data [file Supp_FigureS1-S2.pdf]

Supplementary Data

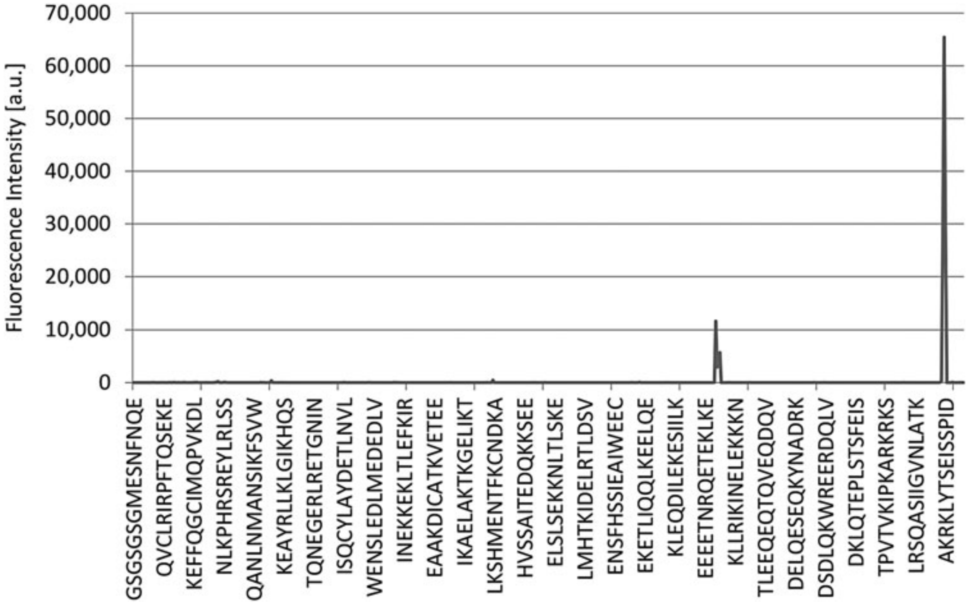

SUPPLEMENTARY FIG. S1. Epitope mapping of monoclonal 10C7 with overlapping peptides representing the full-length KIF20B protein by PEPperPRINT GmbH). Monoclonal 10C7 strongly reacted with a peptide near C-terminus (KLYTSEISSPIDISG), which included the immunizing peptide and weakly with another peptide (ARTQNLKADL QRKEE) toward the N-terminus.

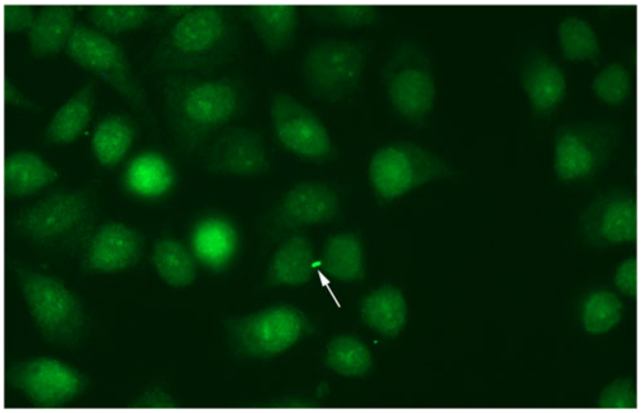

SUPPLEMENTARY FIG. S2. HeLa cells stained with monoclonal 10C7 (1:10 diluted in PBS) show cell-cycle variable staining of nuclei and nucleoli and intense staining of the intercellular bridge (arrow). Fixation/permeabilization: 4% paraformaldehyde/0.2%Triton X-100. PBS, phosphate-buffered saline.
